# A Systematic Composite Service Design Modeling Method Using Graph-Based Theory

**DOI:** 10.1371/journal.pone.0123086

**Published:** 2015-04-30

**Authors:** Arafat Abdulgader Mohammed Elhag, Radziah Mohamad, Muhammad Waqar Aziz, Furkh Zeshan

**Affiliations:** 1 Department of Computer Science, Faculty of Computer Study, International Universiti of Africa (IUA), Khartoum, Sudan; 2 Department of Software Engineering, Faculty of Computing, Universiti Teknologi Malaysia (UTM), Skudai, Johor, Malaysia; 3 Science and Technology Unit, Umm Al-Qura University, Makkah, Saudi Arabia; Tianjin University of Technology, CHINA

## Abstract

The composite service design modeling is an essential process of the service-oriented software development life cycle, where the candidate services, composite services, operations and their dependencies are required to be identified and specified before their design. However, a systematic service-oriented design modeling method for composite services is still in its infancy as most of the existing approaches provide the modeling of atomic services only. For these reasons, a new method (ComSDM) is proposed in this work for modeling the concept of service-oriented design to increase the reusability and decrease the complexity of system while keeping the service composition considerations in mind. Furthermore, the ComSDM method provides the mathematical representation of the components of service-oriented design using the graph-based theoryto facilitate the design quality measurement. To demonstrate that the ComSDM method is also suitable for composite service design modeling of distributed embedded real-time systems along with enterprise software development, it is implemented in the case study of a smart home. The results of the case study not only check the applicability of ComSDM, but can also be used to validate the complexity and reusability of ComSDM. This also guides the future research towards the design quality measurement such as using the ComSDM method to measure the quality of composite service design in service-oriented software system.

## Introduction

Over time the software development has improved using different paradigms, from procedural programming to Object-Oriented Computing (OOC) and Component-Based Computing (CBC) to more recent Service-Oriented Computing (SOC) [1]. SOC paradigm is one of the established paradigms used for building and developing the software products [2–4]. SOC has been applied successfully to develop different types of software systems such as enterprise software systems [5], enterprise information system [4] and distributed embedded real-time systems [6]. SOC, an advancement of CBC and OOC[7], provides many advantages over traditional paradigms such as flexibility, agility, and reusability[6, 7]. However, SOC is quite different from CBC and OOC;as itapplies service as the basic design concept compare to component and class [8], and supports the dynamic composition of services to provide increased reusability. It is important to note that a service is different from a component, because the service functionality is common and not tightly bound to a single client [7, 9]. Moreover, service orientation provides a new level of abstraction i.e., SOC gives and adds the services as a third level of abstraction comparing to maximum two levels of abstraction in traditional paradigms [10]. In brief, the data and codes are encapsulated in procedure as only one level of abstraction in the procedural paradigm[5, 8]. Whereas, the methods in OOC are encapsulated in object-oriented classes, producing two level of abstraction.

SOC is a popular design paradigm for building and developing software systems based on a fundamental and abstract design unit called *service*[4, 10]. A service is “an implementation of stateless, self-contained and well defined pieces of functionality which is published by services provider and can be used by service consumers when building and developing different software systems[11]”. The service design contains interfaces, operations and messages which can operate on dual modes that are published or discovered [2, 6]. Applying services as a fundamental design element of SOC is advantageous in terms of rapid and low cost software development. The service is reusable which allows the construction of systems from already existing applications. Consequently, SOC has become the most suitable paradigm to develop software systems[12].

In service-oriented system the services couldeitherbe atomic or composite[13, 14]. The atomic service refers to a single service that contains some operations and messages to achieve an especial task. A dependency between candidate services describes that a service requires another service or operations for fulfilling its functionality. In other words, the service’s functionality is collected in one service with one interface. In contrast, the composite service is a large service encompassing some atomic services [15]to decrease the complexity of service design by increasing reusability and cohesion and reducing the coupling. The service composition now is a new trend towards developing the software.

Although SOC promised many advantages, still the design structures of service are yet to be defined well. As mentioned in [2], there is no single definition for service-oriented design principles and this makes the concepts of service-oriented design difficult to understand. As a result, the service-oriented software application is regularly designed in an ad hoc manner and success of service oriented design depend on the experience of the software developer [7]. Therefore, the quality of software is negatively affected. In addition, there are numerous quality attributes identified which the service design should fulfill in order to achieve the goals related with the service-oriented application, such as an increased flexibility [2], [16], [17], [18], reusability [9] or maintainability [8]. The principles of designing a service-oriented application that support these quality attributes are reusability, composability, abstraction, cohesion, granularity, loose coupling, design size, discoverability, and autonomy [7], [10], [19], [20], [21]. Moreover, recent literature shows a strong need to propose ways for modeling the composite service design to increase the reusability of the service-oriented systems design and to facilitate the design quality measurement.

Service design modeling is the process of identifying and specifying services and their operations [2, 13, 22]. it is an essential process of service-oriented software development life cycle (SO-SDLC)[23, 24], because the candidate services, composite services, operations and their dependencies are required to be identified and specified first before their design [2, 6, 13, 16, 22]. Thus, the Service-Oriented Design (SOD) is said to be consists of three basic processes i.e., identification, specification and realization[25, 26]. To the best of our knowledge, a systematic service-oriented design modeling method for composite service is still missing, as the existing modeling approaches are either for atomic services or they are based on OOC or CBC. For instance, Perepletchikov, et al. [5, 27] proposed a formal model to represent the service design concept based on a mathematical model. But, this model is for atomic service that does not consider the composite service and lacks in representing the relationships ofcomposite services and their dependencies.

Other authors propose formal models to represent the concept of software design, for example,Briand, et al. [28] proposes a generic model to evaluate the principle properties of software design and the source of metrics. The Briand’s model has been successfully extended by many authors. Neither original model nor the extended models are suitable to be applicable for service-oriented systems [5]. Because, these models are defined based on the particular paradigm; such as OOC and CBC, making them inapplicable to service oriented systems due to their special characteristics.

For these reasons, a new method called ComSDM is proposed to model the concept of service-oriented design using graph-based theorybased on the service composition considerations. Furthermore, the ComSDM method provides the mathematical representation of the components of service-oriented design using the graph-based theory. The ComSDM method is implemented in the case study of smart home to validate the method and to show its suitability for composite service designin both distributed embedded real-time systems (DERTS) and enterprise software system development. The results of the case study not only check the applicability of the ComSDM method,but can also be used to validate the complexity and reusability of the proposed method along with the guidance to continued research. That is using the ComSDM method to measure the quality of composite service design in service-oriented software system.

The rest of the paper is organized as follows: Section 2 contains the methodology to develop the ComSDM method. The ComSDM method is presented in Section 3. Section 4 provides the results ofimplementationof the ComSDM method on the smart home case study. Section 5 provides the validation of the ComSDM method through metrics and discussion is provided in Section 6. Finally, Section 7 concludes the paper.

## Methodology

Research has been done on service-oriented design modeling for atomic service during enterprise software system development. Although, some of the existing service-oriented designmodels are close to this modelingsuch as [5, 27, 29], they do not consider the composite service and are not suitable for DERTS as they lack device considerations and are based on business process modeling. Moreover, these service design modeling approachesare for enterprise software development and the modeling of servicedesign components are done based on mathematical equations.

The existing service-oriented design modeling approaches are derived from a generic software system model proposed by [28], where a software system S is represented as a pair <E, R> of system components and relationships. The extensions are based on the characteristics of the service orientation system. In addition, E is the set of software system components and R is the relationships between software system components representing by (R⊆ E×E). As this model is generic various researchers have successfully extended it to a specific domain or paradigm in order to validate software system based on the specific paradigm characteristics. Neither the original nor the extended models are directly applicable to service-oriented system design due to specific characteristics of this paradigm. This fact is known to the researchers in service-oriented design modeling as reported by [5, 27]. For this reason, these researchers extend the generic model to cover the service-oriented design characteristics, but they consider only atomic serviceswhile ignoring the composite service and service composition. To cover this, a service design modeling methodis proposed,in this work,based on graph theory and the core characteristics of service-oriented paradigm, while keeping the service composition considerations in mind. It is believed and demonstrated that the proposed service designmodeling method is suitable for service design during both DERTS and enterprise software development.

The mostimportant processes in the design of the service-oriented system are service identification and modeling[22]. This work intends to use the previously developed service identification techniques and apply in the ComSDM method to check its validity. In this regard, theexisting service identification approaches are reviewedfrom most famous surveys such as [22, 30–33];in order to select the best one among them and closer to the ComSDM method based on some criteria shared between the selected service identification technique and the ComSDM method. Mohamad, et al.[6]proposes a guideline to identify services in service-oriented system incorporating different criteria such asstep-by-step guideline to identify the service, grouping the operations in services based on task-centric, device consideration, composite service consideration, for developing DERTS and its successful application in smart home case study[6]. Therefore, this work used service identification guideline proposed by Mohamad, et al.[6] to identify candidate services and operations. The selection of this guideline is due to the criteria of the guideline which are most suitable for the ComSDM method. It is clear that the mechanismof the identification technique and the ComSDM method is well-matched as both achieve their goal step-by-step. Furthermore, there are some similarities between ComSDM method and selected service identification technique in many criteria among them: both groups the operations in the services based on task-centric and consider the composite service as well as atomic service. As the ComSDM method is going to consider the enterprise software system and DERTS, the device consideration is shared criteria between these two works along with the successful application in thesmart home case study to check the validity of the work. Consequently,using the selected service identification technique successfully by ComSDM method and its application in the smart home case study proves the suitability of this method for DERTS along with enterprise systems development. The result of services identification and operations candidate using this guideline has been used to apply in ComSDM method.

**Fig 1:**
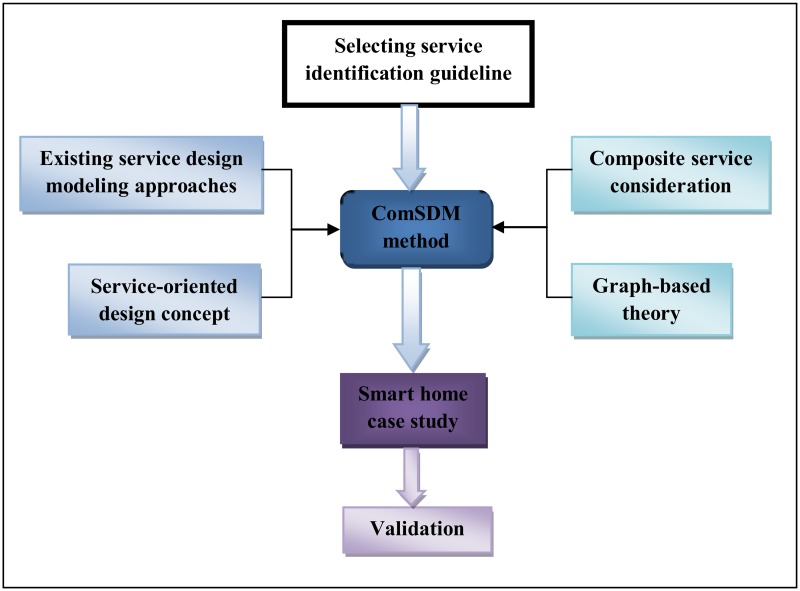
Methodology for deriving the ComSDM method.

In order to have a systematic composite service design modeling method, the existing modeling approachesto service-oriented design and for software development were identified from an extensive search and analysis of the literature. In this work, a systematic composite service design modeling method for service-oriented design is proposed in the following steps. Firstly, the design of service-oriented system is identified and all the components of the design clearly defined, including the system structure, service, composite service, service’s interface, service2010039s functionality and operation’s interface. Secondly, the relationships between the service-oriented design components are defined and new type of relationshipsis identified. Thirdly, the graph-based theorynotationsare used to represent the design components which are extended to cover the missingnotationsin service-oriented design components and its relationships. Fourthly, the processes of the ComSDM method are produced in five steps to show how the method works. Fifthly, the ComSDM method was refined by applying it in the case study of a smart home. Thus, by defyingthe ComSDM method in this fashionmakes it suitable for composite service modeling during service-oriented design. Finally, the ComSDM method was validated by measuring the quality of system design and compared the result with FMSOD. Fig 1 shows the entire methodology for deriving the modeling method.

## The ComSDM Method

As mentioned above, service-oriented design consists of three basic processes, identification, specification and realization. The main contribution of this research work is to identify and propose a definition ofservice-oriented system components covering structural service-oriented design characteristics. In addition,to facilitate in establishing a design modeling method to represent the concept of composite service and service-oriented design components based on graph theory. The ComSDM method is going to give two benefits. Firstly, it describes the design principles and concepts of composite service in service-oriented systems based on graph theory. Secondly, the ComSDM method facilitates proposing measurement to assess the structures of service-oriented design.

### 3.1Design components definition

**Fig 2:**
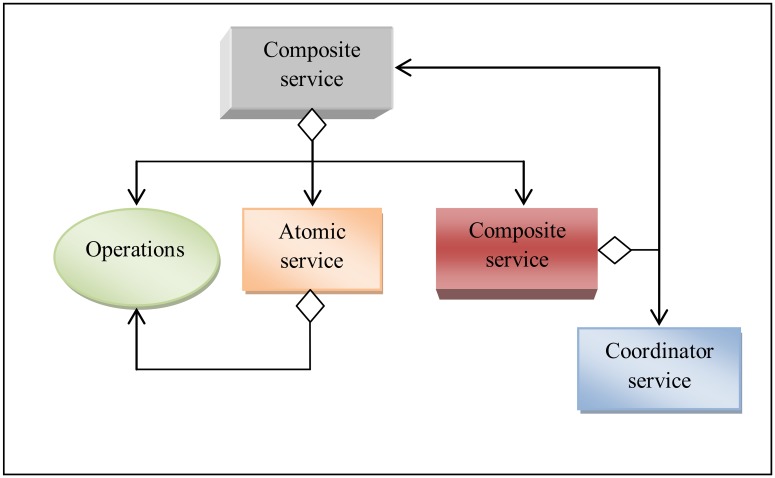
Composite service structure.

**Fig 3:**
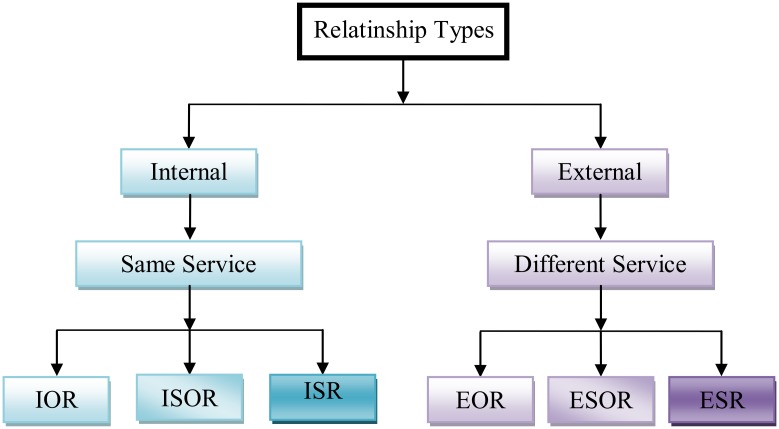
The relationship types.

Service-oriented systems are designed to create a dynamically organized environment for a group of related services and service components that are reusable and composable. In order to propose effective and practical service design modeling many components of service-orientation should be identified and clearly defined. Therefore, components defined in this research are:

- System structure: The system structure (SOS) refers to all the components of service-oriented system as a set of composition design entities. Service-oriented system is aparadigm to build and develop the software applications using clearly-defined functionality from a set of available services with well-defined interfaces while enabling discovery, selection, invocation and composition of services [34]. These entities are a set of services, composite services,service interface, operations, messages and relationships. These entities are discussed as follows:
- Service: The service (S) is an implementation of stateless, self-contained,clearly-defined piece of functionality with well-defined interfaces. It is published by services provider and can be used by service consumers when building and developing different software systems. The functionalities of the service have been provided by the service’s operations or composing other services. The service is reusable which allows the construction of systems from already existing applications. Applying services as a fundamental building block for design of service oriented systems is advantageous in terms of rapid and low cost software development.
- Composite service:The service can be either an atomic service or a composite service. The atomic service is a single service as defined above. The composite serviceis a service constructing from more than one atomicand/or other composite serviceswith one service to coordinate the interactions between the composite service components and the other elements in the system. Fig 2 depicts the structure of the atomic services and composite services.
- Service’s interface: The Service’s Interface (SI) defines the service component to manage the interactions between a service’s functionalities and the external world (other services and operations) by providing or requesting functionalities. The service is designed with interface and operate on published and discovered mode [2]. So, each service should be designed with only one interface that describes the functionalities of the service and how to communicate with it.
- Service’s functionalities: The Service’s Functionalities (SF) mean the tasks that should be achieved by the service. These tasks can be achieved through the set of operations provided by the specific service[5]. Usually, the service needs to compose additional services to accomplish its tasks. Sometimes, the tasks completed by transmitting messagesbetween the operations of the service or between the other operations on different services.
- Operation’sinterface: The Operation’s Interface (OI) defines the parameters of the operation that should be received from communicating components and the messages that sent to other components. Each operation contains a specific interface to communicate with the other service-oriented system components such as services or other operations through their interfaces. This interface is discussed in moredetail below.
- Relationships: The Relationships (R) are the dependencies between the service-oriented system components. The dependencies between services and its components describe that a service requires other services for fulfilling its functionalities or the service invokes the operations of other services. Also, the operations can invoke other operations in the same service or in the other services to fulfill its functionalities. The relationships have been divided into internal and external relationships. So, service-oriented system has six relationship types; three internal and three external relationships. Fig 3 shows the internal and external relationship types.
  - Internal relationships: The internal relationships indicate the internal interaction among the components of a specific service themselves. These are relationship among service’s interface (SI) with operation’s interfaces (OI), the relationship between service’s interfaces in composite service, and relationships among the operation’s interfaces (OIs) themselves. Namely, Internal Service’s and Operation’s Relationships (ISOR), Internal Service’s Relationships (ISR), and Internal Operations Relationships (IOR). For example, one operation can exchange a message between Service Interface with other Operations Interface or Operations Interface among themselves.
  - External relationships: In contrast, the external relationships indicate the external interaction between the service and its components with different service and its components. These are the relationships among the service’s interfaces (SIs) themselves, the relationships among the service’s interface (SI) with the operation’s interfaces (OIs) in different services and the relationships among the operation’s interfaces (IOs) in service S1 with the operation’s interfaces (IOs) in other services S2. These are, External Services Relationships (ESR), External Service’s Interfaces and Operation’s Interfaces Relationships (ESOR), and External Operations Relationships (EOR).

### 3.2 Graph-based theory

Graph-based theory is among the many approaches used for service composition modeling [16]. The graph-based theoryprovides definitions for graph-based, sub graph and graph isomorphism. According to Bunke, et al. [35] a graph G is a 4-tuple *G* = (*V*,*E*,*α*,*β*). Where V is a set of nodes (vertices)which will be used to represent the components of service-oriented system, E is a set of edges linkingthevertices in the graph *E*⊆*V*×*V* and also the edges will be used to represent the relationshipsbetween the components of service-oriented system. The symbol α is a function labeling the nodes *α*:*V*→Σ*v*, and *β*:*V*×*V*→Σ*e* is a function labeling the edges,the (Σ*v* and Σ*e* being the sets of labels that can appear on the nodes and edges). In other words, these functions used to give the vertices and edges the appropriate marks or names. A graph *G*1 = (*V*1,*E*1,*α*1,*β*1) is a sub graph of a graph *G* = (*V*,*E*,*α*,*β*), denoted *G*1*⊆G*,*if V*1*⊆V*, *E*1*⊆E∩(V*1*×V*1),*α*1*(x*) = α(*x*)∀*x*ϵ*V*1 and.*β*1(*x*,*y*) = *β*(*x*,*y*)∀(*x*,*y*) ϵ*E*1.

**Fig 4:**
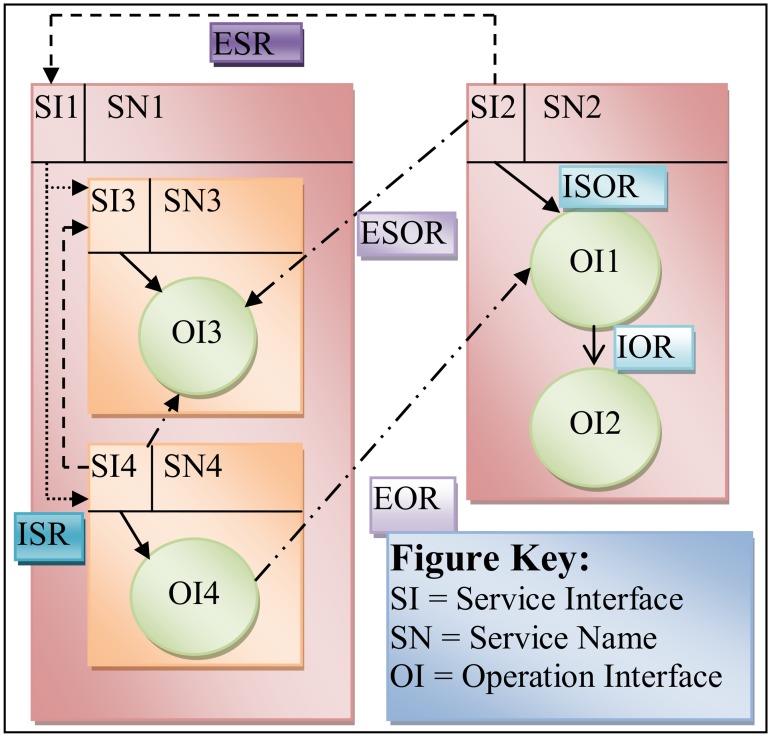
Example for service-oriented system components.

Graph-based representation does not only represent the components of service-oriented design as a graph by vertices, but it also describes the relationships between the service-oriented design components as the edges. Moreover, graph-based representation relies on different levels of representing the service-oriented design components. Service-oriented design representation is divided into three levels: the system level, the service level and the operation level. The representation of these levels within a graph defines the graph nodes and graph edges. Fig 4 shows an example of the components of service-oriented system representing in graph theory by graph G used to build the ComSDM method.

This example introduces a service-oriented system which is represented by graph G and contains two sub-graphs G1 and G2 representing the services SN1 and SN2 respectively. Each sub-graph is symbolized in a rectangle and contains a service interface, service name, operation(s) and set of internal and external relationships. Service SN1 is a composite service contains services SN3 and SN4,represented by a sub-graph G3 and G4 respectively. As illustrated in Fig 4, each service contains a set of operation represented by circle in this modeling, where the name of operation interface is written inside the circle. The relationships between the services and the operations are in different shapes as shown in the Fig 4.

### 3.3 TheComSDM method processes

The processes of the ComSDM method for composite service are describedstep-by-step below to represent the service-oriented system design using graph-based theory.

#### Step 1: Identify the service’s functionalities

The Service’s Functionalities have to be identified first, to achieve the tasks of the service-oriented system. These tasks can be achieved through the set of operations provided by the specific service. In this method the tasks (SF) were identified using previously defined service identification guideline[6], asproposingservice identification technique is outside the scope of this work.

#### Step 2: Identify the candidate operations

In this step, all the candidate operations are identified in a service-oriented system. Formally, the operations O (sf) in service-oriented system are defined as follows:

$$o\in si_{s}\cup S_{s}\cup OI_{s}\cup R_{s}$$ (D1)

For each component used to achieve the services functionalities;let O (sf) be the set of generic operations to achieve the service’s functionalities sf. Also, all operations should have interfaces to describe the set of operation’s parameters. Formally, operation’s interface defined as follows: for each oϵO(sf) let O(s)be the set of generic operation’s interface oi_s._ The operations in service-oriented system have been represented in graph-based notation duringComSDM method by Node as shown in Fig 4.

#### Step 3: Representation of candidate service

In this step, all the candidate services are identified in a service-oriented system. The service is a fundamental building block of service-oriented system that has been represented in the graph-based notation duringComSDM method by sub graph and symbolized by rectangle as shown in Fig 4. Each service contains only one service’s interface; but if and only if the service is composite service and has been constructed from more than one service it contains service’s interfaces for each participated services known by atomic service, set of operations to achieve its functionalities, operation’s interfaces and relationship of services. Formally, a service (s) in service-oriented system is defined as:

$$s=<si_{s},S_{s},OI_{s},R_{s}>$$ (D2)

Given the service-oriented system *SOS* = <*SI*,*S*,*OI*,*R*>, a services = <*si*_*s*_,*S*_*s*_,*OI*_*s*_,*R*_*s*_> is a service of SOS if and only if *si*_*s*_ ϵSI⋂(*S*_*s*_⊆*S*⋂*OI*_*s*_⊆OI⋂*R*_*s*_⊆*R*⊆(*si*_*s*_× *OI*_*s*_)) ⋂(*si*_*s*_⋃*S*_*s*_⋃*OI*_*s*_⋃*R*_*s*_〈〉*s)*. The service membership has been represented by the “〈〉” symbol[5]. Also, some of the components of the service-oriented system could be absent from the service or the system. As such, the matching set of components would be empty as indicated by∅ symbol. For example, the following Equation provides the design of the service SN2 in service-oriented system as shown in Fig 4.

$$sn2=<si_{sn2},S_{sn2},OI_{sn2},R_{sn2}>$$

The service sn2 is a single service and it is designed with one service and two operations and there is no any composite service. The service sn2 consists of one service interface *si*_*sn*2_ = {*si* 2}, this is no composite service because sn2 is a single service *S*_*sn*2_ = ∅, also sn2 contains two operation interfaces *OI*_*sn*2_ = {*oi*1, *oi*2}and an edge set that represents the relationships between the component of SN2 service and the other service components R_*sn*2_ = {(*si*2,*oi*1),(*si*2,*si*1),(*si*2,*oi*3),(*oi*1,*oi*2),(*oi*4,*oi*1)}. Following is the complete representation of service sn2:

$$sn2=<si_{sn2},S_{sn2},OI_{sn2},R_{sn2}>=<\{si2\},\phi ,\{oi1,oi2\},\{(si2,oi1),(si2,si1),(\text{si}2,\text{oi}3),(\text{oi}1,\text{oi}2)\}>$$

#### Step 4: Representation of the relationships

The relationships (R) have been represented by edgesduringComSDMmethod using graph-based theory. More definitions and representation of these relationships by graph-based methods are shown as follows:

- Internal Service’s and Operation’s Relationships (ISOR): the relationship among the service’s interface with operation’s interfaces in the same service. ISOR has been represented by solid arrow symbol in the graph-based notation during ComSDM method. Formally, ISOR is defined as:
  $$ISOR(s)=\{(si_{s},oi_{s})\in R_{s}|R_{s}\subseteq (SIOI)\cap si_{s}\in SI\cap oi_{s}\in OI\cap (si_{s},oi_{s})\in S_{s}\}$$ (D3.1)
  For example, in Fig 4 the *ISOR*(*SOS*) = {(*si*2,*oi*1),(*si*3,*oi*3),(*si*4,*oi*4)}.
- Internal Service’s Relationships (ISR): the relationship among the service’s interfaces themselves in the composite service. ISR has been represented by round dot arrow symbol in the graph-based notation during ComSDM method. Formally, ISR is defined as:
  ISR(s)={(si1,si2)∈Rs|Rs⊆(SISI)∩(si1,si2)∈SIs∩(s1,s2)∈s∩si1∈SIs∩si2∈(SIs-si1)∩(s⊇(si1∩si2)} (D3.2)
  For example, in Fig 4 the *ISR*(*SOS*) = {(*si*1,*si*3),(*si*1,s*i*4)}.
- Internal Operations Relationships (IOR): the relationship between the operation’s interfacesthemselves in the same service. IOR has been represented by solid open arrow symbol in the graph-based notation during ComSDM method. Formally, IOR is defined as:
  $$\text{IOR}(\text{s})=\{({\text{oi}}_{1},{\text{oi}}_{2})\in {\text{R}}_{\text{s}}|{\text{R}}_{\text{s}}\subseteq (\text{OIOI})\cap ({\text{oi}}_{1},{\text{oi}}_{2})\in {\text{OI}}_{\text{s}}\cap ({\text{o}}_{1},{\text{o}}_{2})\in \text{s}\cap ({\text{o}}_{1}\neq {\text{o}}_{2})$$ (D3.3)
  For example, the *IOR*(*SOS*) = {(*oi*1,*oi*2)}in Fig 4.
- External Services Relationships (ESR): the relationship among the service’s interfaces themselves. This relationship indicated that one service in service-oriented system uses the other service to achieve its functionalities. However, ESR has been represented by dash arrow symbol in the graph-based notation during ComSDM method. Formally, ESR is defined as:
  $$ESR(s)=\{(si_{1},si_{2})\in R_{s}\text{|}R_{s}\subseteq (SISI)\cap si_{1}\in SI_{s}\cap si_{2}\in (SI-SI_{s})\}$$ (D3.4)
  For example, the *ESR*(*SOS*) = {(*si*2,*si*1),(*si*4,*si*3)} in Fig 4.
- External Service’s Interfaces and Operation’s Interfaces Relationships (ESOR): the relationship among the service’s interface with the operation’s interfaces in different services. This relationship may be from the service interface to operation interface or vice versa. So, ESOR has been represented by long dash and dot arrow symbol in the graph-based notation during ComSDM method. Formally, ESOR is defined as:
  ESOR(s)={(a,b)∈Rs|Rs⊆(SIOI∪OISI)∩((a∈SI⇔b∈OI)∪(a∈OI⇔b∈SI))∩(a∈(SIs∪OIs)∩b∈(SI-SIs∪OI-OIs))} (D3.5)
  For example, the following is the representation of the *ESOR*(*SOS*) = {(*si*2,*oi*3),(*si*4,*oi*3)}in Fig 4.
- External Operations Relationships (EOR): the relationships among the operation’s interfaces in service *s*_1_with the operation’s interfaces in other services *s*_2._ EOR has been represented by long dash and double dot arrow symbol in the graph-based notation during ComSDM method. Formally, EOR is defined as:
  $$EOR(s)=\{(oi_{1},oi_{2})\in R_{s}|R_{s}\subseteq (OIOI)\cap (oi_{1},oi_{2})\in OI\cap (oi_{1}\in OI_{s}\cap oi_{2}\in (OI-OI_{s}))\}$$ (D3.6)

The following is the representation of the EORwhich is shown in Fig 4: *EOR*(*SOS*) = {(*oi*4,*oi*1)}.

#### Step 5: Representation of thesystem structure

The system structure in service-oriented system is a representative subset comprising the total subset that represents the components of the services in service-oriented system. The graph has been used to represent the service-oriented system in graph-based theory during ComSDM method. Formally, system structure (SOS) defined as:

SOS=〈SI,S,OI,R〉 (D4)

Where SI is a set of all Service’s Interfaces in SOS; S is a set of all services in SOS; OI is a set of all operation’s interfaces in SOS; and R is a set of all relationships in SOS. The following is the representation of the service-oriented system which is shown in Fig 4:

SOS=<SI,S,OI,R>=<{si1, si2, si3, si4}, {sn1, sn2, sn3, sn4}, {oi1, oi2, oi3,oi4},{(si2, oi1),(si2, si1),(si2, oi3),(oi1, oi2),(oi4, oi1),(si1, si3),(si1, si4),(si3, oi3),(si4, oi4),(si4, oi3)}>

## Implementation of the Modeling Method

The ComSDM method for service-oriented design provided in section 3isimplemented for the smart home system case study, as introduced in[6],to verify and check the applicability of this method.

### Step 1: Identify the service’s functionalities

For this step, the smart home system tasks have to be identified to achieve the service’s functionalities. These tasks can be achieved through the set of operations provided by the specific service. The identified tasks (FS) are as listed below:

- While the user attends a phone call then the television (TV) volume automatically decreases, and volume increases when the user finishes.
- The lights become full automatically when the TV is turned off and dim while the user is watching the TV.
- The air conditioner (AC) speed depends-on the Temperature Sensor reading and without human intervention becomes slow or fast.
- During the food is cooking the Oven reads the radio frequency identification (RFID) tag to cook and when the food is finished an appropriate message is sent to user on cell phone and TV.
- Fridge checks the weight of food item, orders to retail store and sends an email to the user.
- Fridge checks the expiry date of food items and sends a message to the user if the food is expired.

### Step 2: Identify the candidate operations

**Fig 5:**
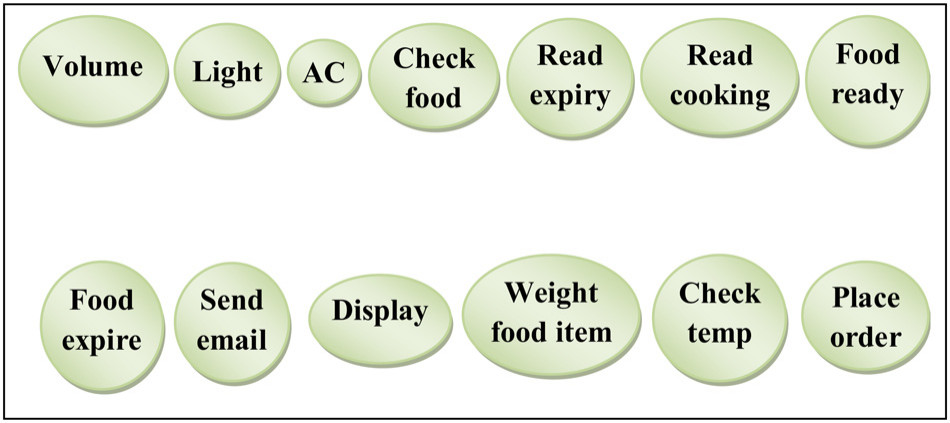
Candidate operations in smart home system.

After, applying the guideline for smart home case study [6] the set of candidate operations for the entire systemwas identified based on the tasks highlighted in Step1. These candidate operations and its Interfaces are shown in Table 1. The candidate operations have been represented by Nodes in graph-based during ComSDM method. In case of the smart home there were thirteen operations, each of which was represented by a node using circle shapes in graph-based during ComSDM method as shown in Fig 5. Moreover, the operations interfaces names have been written inside the node to facilitate defining the relationships and grouping the identified operations into logical units to construct the services.

**Table 1:** Candidate operations in smart home system.

#	Candidate Operations	Operations Interfaces
1	Low/High Volume	Volume
2	Low/High Light	Light
3	Low/High AC	AC
4	Check the expiry date on the food item	Check food
5	Read expiry date on the food item	Read expiry
6	Read Cooking Instructions on the food item	Read cooking
7	Send SMS to user’s cell phone—Food ready	Food ready
8	Send SMS to user’s cell phone—Food expire	Food expire
9	Send message to user email account—Food empty	Send email
10	Display message to TV—Food ready	Display
11	Check the weight food item	Weight food item
12	Check the temperature	Check temp
13	Order the food item from the retail store	Place order

The following is the representation of the ComSDM method equation for the operations interfaces of the smart home system case study which is shown in Fig 5:

OI(SH)={Weight food item, Send email, Place order, Volume, Read cooking, Food ready,Food expire, Check food, Read expiry, Display, Ac, Check temp, Light}

### Step 3: Represent candidate service

In this step, all the candidate operations identified in above step were grouped together centered on tasks for representing the candidate services in the smart home case study. Thecandidate services areshown in Table 2.

**Table 2:** Candidate services in smart home system.

#	Services Names	Services Interface	Operations Interfaces
1	Volume	VolumeI	Volume
2	Light	LightI	Light
3	AC	AcI	AC
4	Food	FoodI	Check food, Read expiry
5	Read	ReadI	Read cooking
6	Send	SendI	Food ready, Food expire
7	Email	EmailI	Send email
8	Display	DisplayI	Display
9	Weight food item	WeieghtI	Weight food item
10	Check	CheckI	Check temp
11	Place	PlaceI	Place order

Some services are related together to achieve a specific task, so they were combined in a composite service. To coordinate the composite services a control service was added in each composite service. The *AC* and *Check* services belonged to *Controlling Temperature* task, therefore they were combined in a new composite service named *Control* service. The *Email*, *Place* and *Weight* services related to the *Place Order Food* to retail store, so these services were grouped together into a composite service named *Order* service. The *Cook* service is a new service that belonged to the task of *Cooking Food*. The *Display* service was composed into a *Cook* service. In addition, the *Cook* service invoked the *Cooking* operation from *Read* service and *Food* ready operation from *Read* service.The *Cook* service monitoring the food using the operation *Read* cooking, when the food is readyan SMS will send by the operation *Food* ready to the user to inform him the food is ready using *Display* service to change the light state. As a result, the list of candidate services is updated to introduce new services in the list and add the new services interfaces. Consequently, a new list of composite services was obtained as shown in Table 3.

**Table 3:** Composite services in smart home system.

#	Candidate Services	Services Interface	Composite Services
1	Volume		-
2	Light		-
3	Cooking	CookI	Coordinate, Display.
4	Order	OrderI	Coordinate, Weight food item, Email, Place
5	Food		-
6	Control	ControlI	Coordinate, AC, Check
7	Send		-

**Fig 6:**
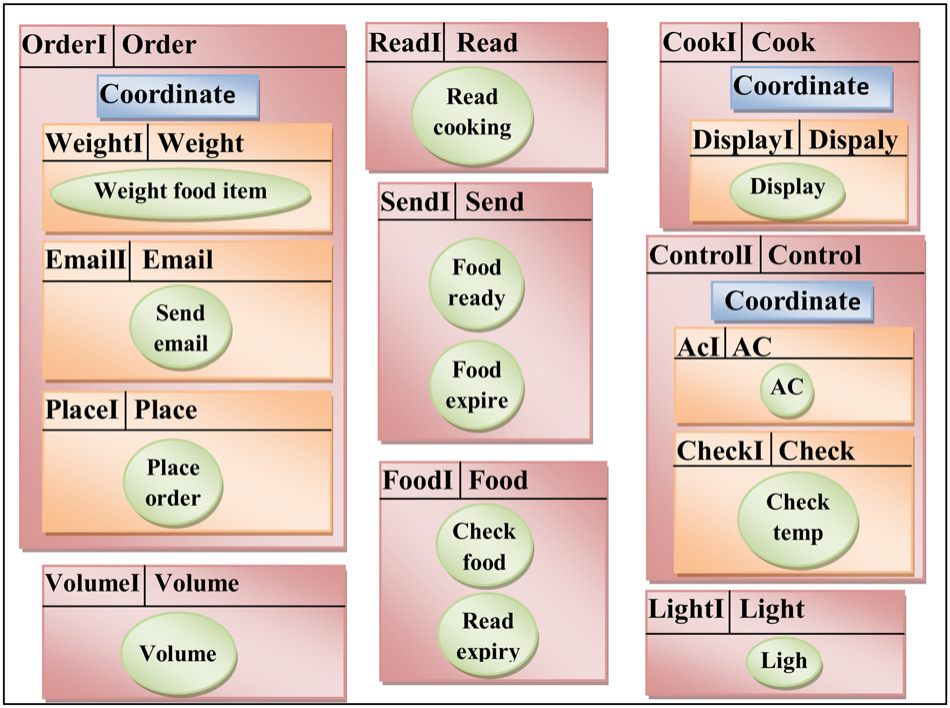
Candidate services in smart home system.

The candidate services have been represented by a sub-graphin graph-based notation duringComSDM method. Fig 6 shows the representation of these services in graph-based during ComSDM method.

Following is the representation of the ComSDM method equation for the services interfaces in a smart home case study which is shown in Fig 6.

*SI (SH) = <{OrderI, WeightI, EmailI, PlaceI, ReadI, CookI, DisplayI, SendI, FoodI, VolumeI, ControlI, AcI, CheckI, LightI}.*

Following is the representation of the ComSDM method equation for the services in smart home case study in the whole system which is shown in Fig 6.

*S (SH) = {{Order, coordinate, Weight, Email, Place}, read, {Cook, Coordinate, Display}, Send, Food, Volume, {Food Ordering, Coordinate, Ac, Check}, Light}, {Weight food item, Send email, Place order, Volume, read cooking, Food ready, Food expire, Check food, Read expiry, Display, Ac, Check temp, Light}.*

Following is the representation of the ComSDM method equation for the services in the smart home case study which is shown in Fig 6.

Secondly, for each service:*s(Order) = <siOrder*, *SOrder*, *OIOrder*, *ROrder> = <{OrderI*, *WeightI*, *EmailI*, *PlaceI*}, {*coordinate*, *Weight*, *Email*, *Place}*, {*Weight food item*, *Send email*, *Place order*}, {(*OrderI*, *coordinate)*, *(OrderI*, *WeightI)*, *(OrderI*, *EmailI)*, *(OrderI*, *PlaceI)*, *(WeightI*, *Weight food item)*, *(EmailI*, *Send email)*, *(PlaceI*, *Place order)*, *(EmailI*, *PlaceI)*, *(PlaceI*, *Send email)}*.

### Step 4:Identify the relationships

In this step all the relationships in the service-oriented system were identified and represented by the edges in graph-based during ComSDM method. The following is the representation of the ComSDM method equation for the relationship types in smart home case study which is shown in Table 4.

**Table 4:** Relationship types in smart home system.

#	Relationship types	Service Interface	Service Interface	Operation Interface	Operation Interface
1	ISR	OrderI	Coordinate		
2	ISR	OrderI	WeightI		
3	ISR	OrderI	EmailI		
4	ISR	OrderI	PlaceI		
5	ISR	CookI	Coordinate		
6	ISR	CookI	DisplayI		
7	ISR	ControlI	Coordinate		
8	ISR	ControlI	AcI		
9	ISR	ControlI	CheckI		
10	ISOR	WeightI			Weight food item
11	ISOR	EmailI			Send email
12	ISOR	PlaceI			Place order
13	ISOR	DisplayI			Display
14	ISOR	AcI			Ac
15	ISOR	CheckI			Check temp
16	ISOR	VolumeI			Volume
17	ISOR	ReadI			Read cooking
18	ISOR	SendI			Food ready
19	ISOR	SendI			Food expire
20	ISOR	FoodI			Check Food
21	ISOR	LightI			light
22	IOR			Check food	Read expiry
23	ESR	DisplayI	LightI		
24	ESR	CookI	ReadI		
25	ESR	EmailI	PlaceI		
26	ESOR	PlaceI			Send email
*27*	*ESOR*		*DisplayI*	*Food ready*	
28	ESOR	FoodI			Food expire
29	EOR			Check Food	Read cooking

*ISR (SH) = {(OrderI, Coordinate), (OrderI, WeightI), (OrderI, EmailI), (OrderI, PlaceI), (CookI, Coordinate), (CookI, DisplayI), (ControlI, Coordinate), (ControlI, AcI), (ControlI, ChechI)}.*

*ISOR (SH) = {(WeightI, Weight food item), (EmailI, Send email), (PlaceI, Place order), (VolumeI, Volume), (ReadI, Read cooking), (SendI, Food ready), (SendI, Food expire), (FoodI, Check food), (DisplayI, Display), (AcI, Ac), (CheckI, Check temp), (LightI, Light)}.*

*IOR (SH) = {(Chech food, read expiry)}.*

*ESR (SH) = {(DisplayI, LightI), (CookI, ReadI), (EmailI, PlaceI)}.*

*ESOR (SH) = {(PlaceI, Send email), (DisplayI, Food ready)}.*

*EOR (SH) = {(Check food, Read cooking)}.*

However, the relationships among the service-oriented system components in the smart home case study are listed in Table 4.

### Step 5: Representing system structure

**Fig 7:**
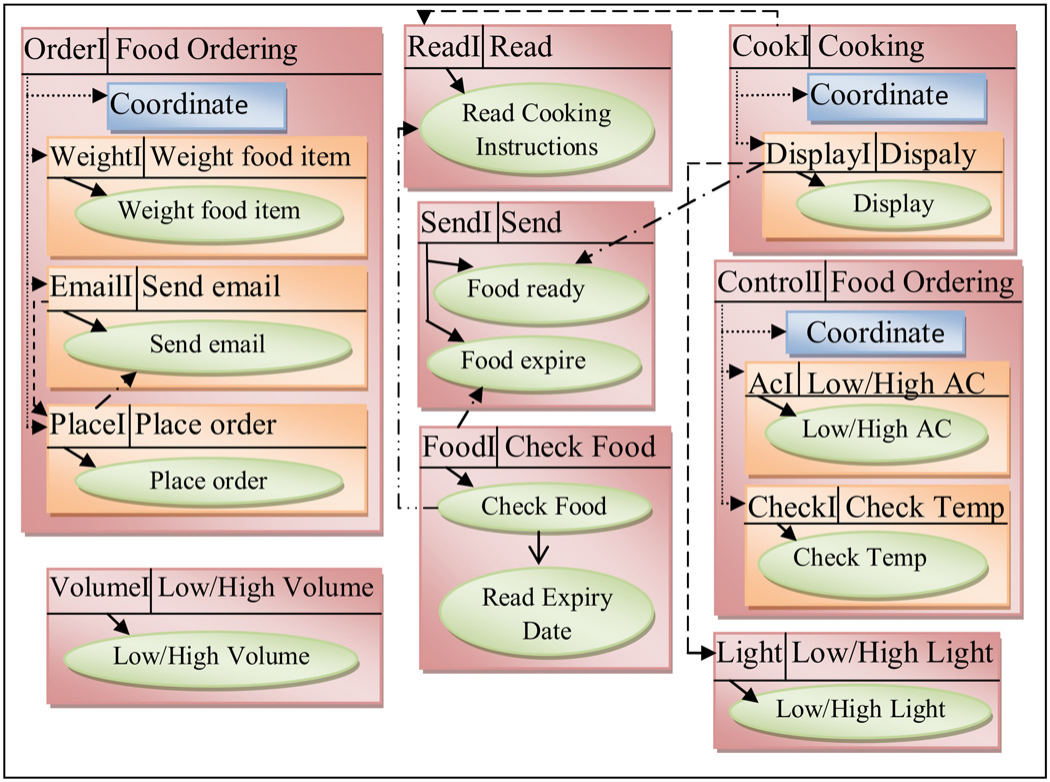
Complete graph of applying the ComSDM method for smart homes.

The system structure in service-oriented system is proposed by combining all the components of the service-oriented system. Fig 7 shows the system structurerepresented by the full graph in graph theory after representing the service, operations and their relationships in ComSDM method.

Following is the representation of the ComSDM method equation in completesmart home system case study which is shown in Fig 7:

*SOS (SH) = <SISH, SSH, OISH, RSH> = <{OrderI, WeightI, EmailI, PlaceI, ReadI, CookI, DisplayI, SendI, FoodI, VolumeI, ControlI, AcI, CheckI, Light, {{Order, coordinate, Weight, Email, Place}, read, {Cook, Coordinate, Display}, Send, Food, Volume, {Food Ordering, Coordinate, Ac, Check}, Light}, {Weight food item, Send email, Place order, Volume, read cooking, Food ready, Food expire, Check food, Read expiry, Display, Ac, Check temp, Light}, {(WeightI, Weight food item), (EmailI, Send email), (PlaceI, Place order), (VolumeI, Volume), (ReadI, Read cooking), (SendI, Food ready), (SendI, Food expire), (FoodI, Check food), (DisplayI, Display), (AcI, Ac), (CheckI, Check temp), (LightI, Light),(OrderI, Coordinate), (OrderI, WeightI), (OrderI, EmailI), (OrderI, PlaceI), (CookI, Coordinate), (CookI, DisplayI), (ControlI, Coordinate), (ControlI, AcI), (ControlI, ChechI),(Chech food, read expiry), (DisplayI, LightI), (CookI, ReadI), (EmailI, PlaceI), (PlaceI, Send email), (DisplayI, Food ready),(Check food, Read cooking)}}>.*

## Validation of the ComSDM Method

**Fig 8:**
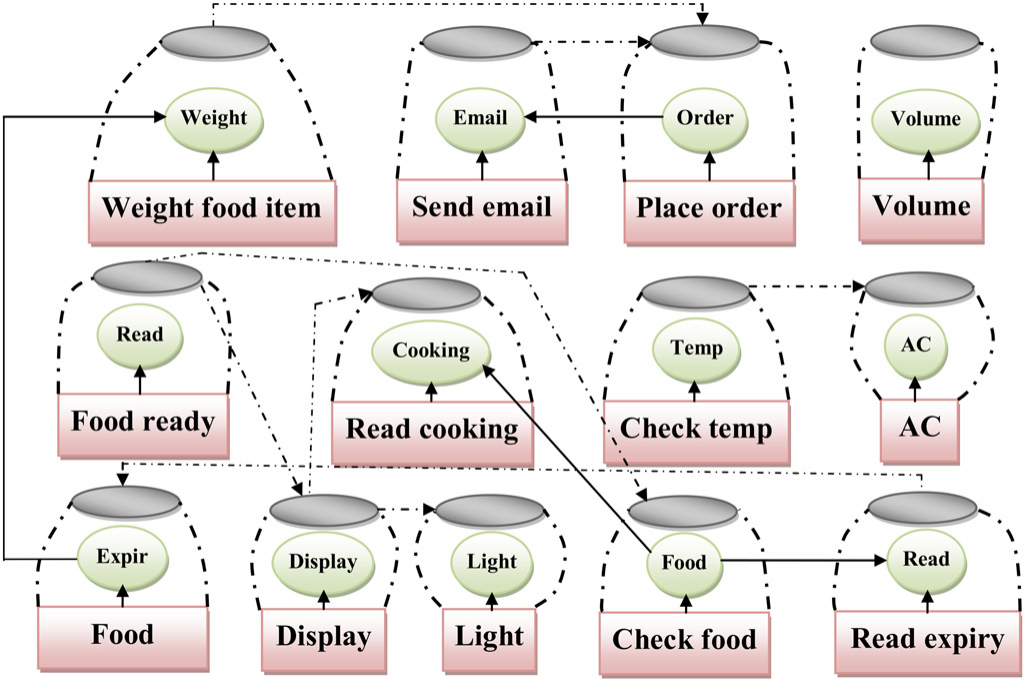
Application of FMSOD to smart home case study.

After applying the ComSDM method successfully in smart home case study, this step is established to validate the ComSDM method through assessing and evaluating the quality of software design. In accordance with the goal of SOC, the ComSDM method in this paper for modeling the composite service reduced the complexity and increased the reusability of service-oriented design. To assess the quality of ComSDM method the metrics for measuring the complexity [36] and reusability [10] for service-oriented design are selected. Coupling and cohesion considered as the main factors to measure the complexity and reusability of software system. Coupling is the interactions between the services and its components in service-oriented design. Whilst, the cohesion estimates the degree to which the components of service-oriented system belong together. The result of ComSDM method is compared with FMSOD[5, 27]. FMSOD is a model for modeling the service-oriented design whichis defined in Perepletchikov, et al. [27] and extended from [5]. FMSOD considers the service as main concept which contains the elements of service-oriented system and each service is a basic service. FMSOD defines the interactions between the services and system elements and also propose some metrics to measure the quality of design to predict the maintainability of the system, FMSOD is the best and closest model to compare with the ComSDM method. The FMSOD is applied to the smart home case study to check the quality of both the ComSDM method and FMSOD. Fig 8 shows the application of FMSOD to thesmart home case study. Following subsections provide the detail of using complexity and reusability metrics with the application of proposed method and FMSOD in the case study.

### 5.1 Number of services and operation

The Number of Services (NS) and Number of Operations (NO) are two simple metrics used to count the number of services and operations in service-oriented system [36]. These two metrics are the first step to calculate the complexity of service-oriented system [37]. The NS and NO of the case study for the ComSDM method and FMSOD are presented in Table 5.

**Table 5:** NS and NO for the proposed method and FMSOD.

Description	Metrics	Proposed method	FMSOD
Number of services	$\text{NS}\left(\text{SH}\right)=\sum_{\text{s}\in SH}\text{s}$	8	13
Number of operations	$NO\left(SH\right)=\sum_{s\in SH}NO\left(s\right)$	13	13
Total	*NS*(*HS*)*+NO*(*SH*)	21	26

As shown in Table 5, the NS identified through FMSOD are more than those identified by the ComSDM method. This result can be considered as the first indicator that the ComSDM method reduces complexity as compared to FMSOD when applying them in the same case study.

### 5.2 Provider and Consumer

The provider is the service or operation that provides functionalities for other services or operations. Whereas, the consumer is the service or operation which invoked the functionalities provided by the providers [36]. The number of providers and consumers for the ComSDM method and FMSOD are shown in Table 6. The result of these metrics is used to calculate the coupling and cohesion metrics in Subsection 5.3 and 5.4.

**Table 6:** Number of providers and consumers for the proposed method and FMSOD.

Description	Metrics	Proposed method	FMSOD
Number of providers	*P* = {(*s*,*o*)ϵ*P*|(*s*ϵ*S*)⋂(*o*ϵ*O*)⋂(*s*^*o*)≠∅⋂(*s*,*o*)ϵ*R*⋂*R is In*}	27	26
Number of consumers	*C*(*p*){(*s*,*o*)ϵC|(*s*ϵ*S*)⋂(*o*ϵ*O*)⋂(pϵP)⋂((s⋃o)⋂p)ϵ*R*⋂*R is Out*}	26	25

### 5.3 Coupling metrics

**Fig 9:**
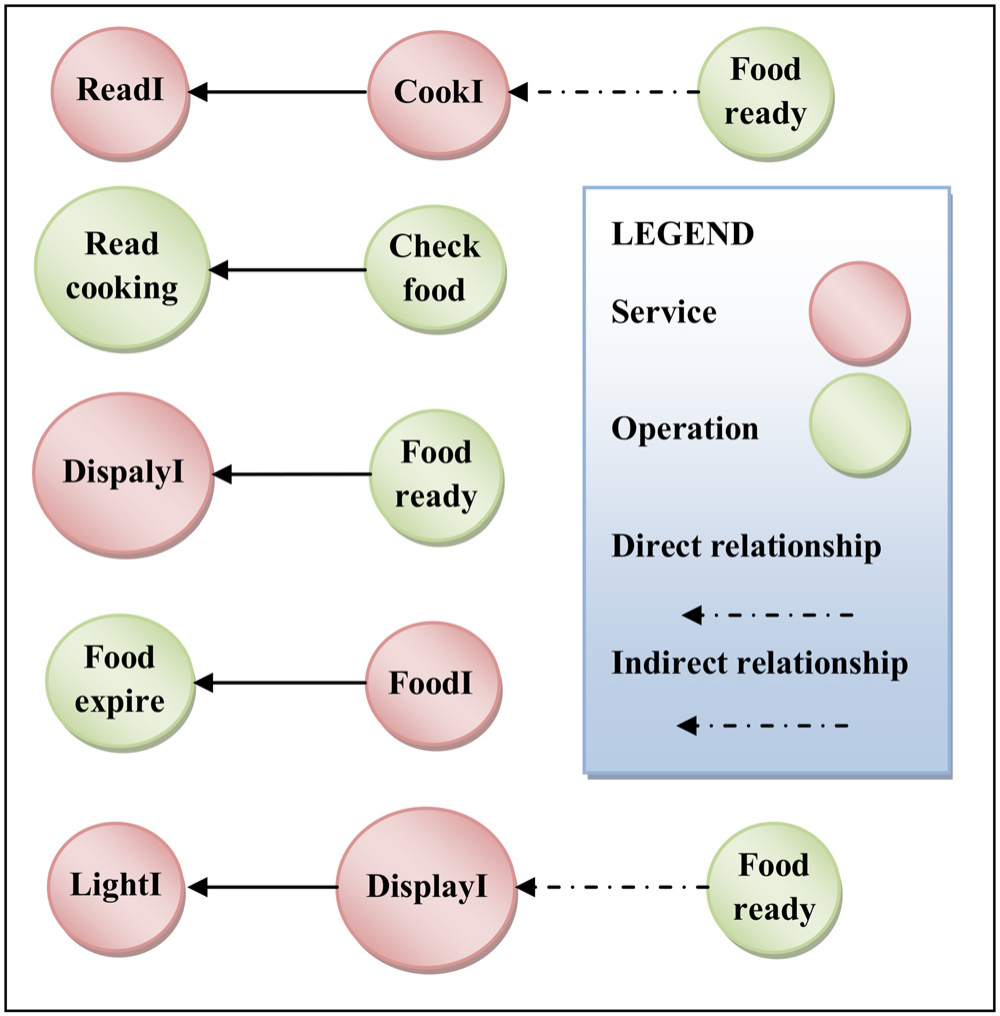
Direct and indirect coupling of smart home in the ComSDM method.

**Fig 10:**
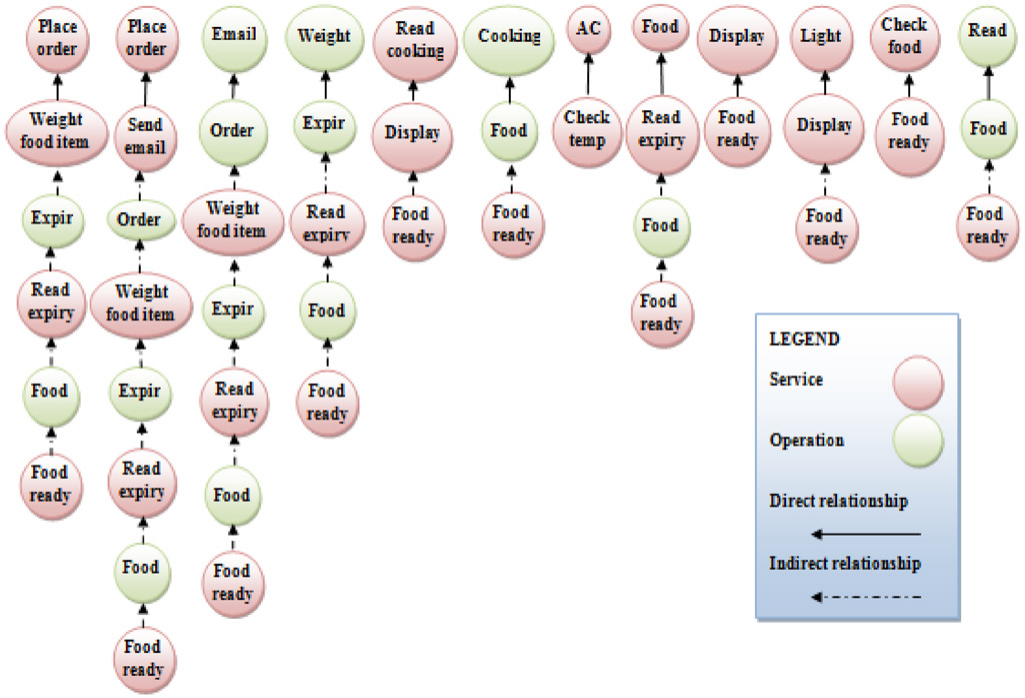
Direct and indirect coupling of smart home in FMSOD.

Mohammed Elhag and Mohamad, et al.[36] proposes two metrics to calculate coupling in service-oriented design which are direct and indirect metrics. Direct coupling is derived metric to calculate the direct interactions in service-oriented design. Whilst, indirect coupling measures the indirect interactions between the services and operations in service-oriented design. Figs 9 and 10depict the direct and indirect coupling of the smart home case study using the ComSDM method and FMSOD, respectively. To calculate the direct and indirect coupling the metrics proposed inMohammed Elhag and Mohamad, et al.[36] are used. Table 7 shows the calculation of direct and indirect coupling for both ComSDM method and FMSOD in the smart home case study. Fig 9 shows that five services are directly coupled and two indirectly coupled when the ComSDM method is applied in the case study. Whilst, Fig 10 shows that twelve services are directly coupled and twenty-four indirectly coupled when FMSOD is applied in the case study. The results show that the system elements in the ComSDM modeling method are loosely coupled as compared by FMSOD. Although, the coupling metrics give an indicator for the interactions between the elements of service-oriented system, but the interpretation of these results depend on the size of the system. In the other words, the results of coupling metrics are numbers and could not be interpreted by itself because these numbers depend on the size of the system. For instance, if the coupling in the system is five this result depends on the size of this system when the system contains 100 services and operations this result are good; whilst, if the system contains 5 services means this system is loosely coupled. Consequently, the coupling factor metric is used to calculate the values of coupling according to the size of software systems. The result of applying this metric for both ComSDM method and FMSOD in smart home is shown in Table 7. The values of the coupling factor indicate that ComSDM method is more loosely coupled than FMSOD.

**Table 7:** Coupling metrics for the proposed method and FMSOD.

Description	Metrics	Proposed method	FMSOD
Direct coupling	DC(*p*) = C(*p*)	5	12
Indirect coupling	$IC\left(p\right)=DC\left(p\right)+\sum_{c\left(p\right)\in P}IC\left(c\left(p\right)\right)$	2	24
Total coupling	DC(*p*)+*IC*(*p*)	7	36
Coupling factor	$CopF(p)=\frac{IC(p)}{f^{2}-f}$//*f = NS(SH)+NO(SH)*	0.02	0.06

### 5.4 Cohesion metrics

The cohesion metric and cohesion factor metric are two metrics used to calculate the cohesion in the smart home case studywhen applying the ComSDMmethod and FMSOD. The result of these metrics is the other indicator to represent the service-oriented system complexity. Table 8 shows the values of using cohesion and cohesion factor metrics for the ComSDMmethod and FMSOD in the case study.

**Table 8:** Cohesion metrics for the proposed method and FMSOD.

Description	Metrics	Proposed method	FMSOD
Total cohesion	*CM*(*s*) = {*c*(*p*)|(*c*ϵ*C*)⋂(*p*ϵ*P*)⋂(*c*⋂*p*)ϵ*s*}	21	13
Cohesion factor	$CohF=\sum_{s\in SH}\frac{CM\left(s\right)*\left(l^{2}-l\right)}{f^{2}-f}$// *l = NS(s) + NO(s) // f = NS (SH) + NO (SH)*	0.95	0.04

The result in Table 8 shows the ComSDMmethod is more coherent than the FMSOD.

### 5.5 Complexity metrics

The services in service-oriented system should be designed in a way through which the total complexity of the system is reduced. The coupling and cohesion metrics are used to calculate the complexity of the ComSDMmethod and FMSOD. Table 9 shows the results of complexity metric, complexity factor and total complexity metric for both the ComSDMmethod and FMSOD.

**Table 9:** Complexity metrics for the proposed method and FMOSD.

Description	Metrics	Proposed method	FMSOD
Complexity	$TCM(s)=\frac{IC(s)+NS(s)+NO(s)}{CM(s)}$	10.25	25
Complexity factor	$ComF(s)=\frac{CopF(s)}{CohF(s)}$	0.02	1.5
Total complexity	$TCM\left(SH\right)=\sum_{s\in SH}TCM\left(s\right)\text{*}ComF\left(s\right)$	2.2	37.5

The results clearly show that the ComSDMmethod reduces the complexity of service-oriented designas compared with FMSOD.

### 5.6 Reusability metrics

The services in service-oriented system should be designed in a way through which the reusability of the system is increased. The reusability in service-oriented system is affected by two factors which are the direct consumers for the service and the degree of cohesiveness of the operations in the service [10]. The services have less direct interactions with other service components and higher cohesiveness between its operations are more reusable. Therefore, the direct coupling metric is used to calculate the direct consumers of each service for the ComSDMmethod and FMSOD; and reusability factor to compare the reusability values with the system size by measuring the cohesion of operations. Table 10 shows the result of reusability metric and reusability factor.

**Table 10:** Reusability metrics for the proposed method and FMOSD.

Description	Metrics	Proposed method	FMSOD
Reusability	DC(p) = C(p)	5	12
Reusability factor	$ResF=\frac{CM(SH)}{DC(SH)}$	4.2	1.08

The results show that the ComSDMmethod has less direct consumers and more coherent than the FMSOD. Therefore, the proposed method is more reusable than FMSOD.

## Related Works and Discussion

The previous works on service-oriented modeling [3, 27, 29, 38, 39]do not consider the design of composite service because these are for modeling atomic service.However, considering the composite service in this modeling method lead to decrease the number of interactions among different services by combining them in one big service. Consequently, the reusability of service-oriented software system increased and the complexity reduced due to decrease the coupling between the services and increase in the cohesion service elements. Perepletchikov, et al. [5, 27] proposes a formal mathematical model for service-oriented design and all artifacts are clearly identified mathematically in this model. Furthermore, the relationships are defined between the components of the service-oriented system. But, the components of the composite service are not considered in this model and also the relationships among the composite services are absent. However, this paper proposes a new method for modeling composite service design using graph-based theory to represent the componentdesign of service-oriented system by graph theory notations. Moreover, the components of composite service design are clearly identified and all the relationships defined especially for composite services. The graph theory notations are extended to cover all composite service design components and the relationships between them.

The ComSDM method for modeling composite services used the service identification technique proposed by Mohamad, et al.[6] and implemented successfully in thesmart home case study. The resultschecked theapplicability ofComSDM method andconfirmed that this method is suitable for DERTS along with enterprise software system development. The results of the case study used also to validate the complexity and reusability of the ComSDM method using the existing metrics for measuring the quality of service-oriented design. The results compared with FMSOD[5, 27] in order to show the better quality of ComSDM methodin term of complexity and reusability of the systems. The result shows the ComSDM method is reduced the complexity of system design and increased the reusability as compared toFMSOD. These results come,because this method gives special attention to model step-by-step the service-oriented design considering the composite services to reduce the external interactions between services. In addition, the metrics result shows the ComSDM method is loosely coupled and more cohesive than FMSOD. The loose coupling and cohesiveness of the services in the ComSDM method lead to reduce the complexity and increase the reusability of service-oriented system,thus confirming that this method adhere to the concept of service-oriented design. Also, the identified services in the ComSDM method are fewer as compared with FMSOD, this givesanother indicator to reduced complexity and coupling and increased reusability and cohesion.

The evaluation of quality attributes for the software system and service-oriented system is very essential and gained much attention among researchers [40–42]. The ComSDM method can be used to guide the continued research, such as proposing measurement to evaluate the quality of composite service design in service-oriented software system. Ding et al. [41] propose a new approach for assessing and evaluating the trustworthiness of selected service in cloud computing and service-oriented computing. This approach assesses the quality of existing services to select among them the best one according to its availability and performance. However, itdoesn’t assess the quality of service in the early stage of the software development life cycleparticularly at design time. Ding et al. [43], proposes a new framework for evaluating the trustworthiness of cloud computing by combing andcomposing the quality of service and customer perspective. The proposed framework named CSTrust, is succeed to design objective measurement along with subjective measurement of cloud service. The quality of a service-oriented product can be measured when the software product has been developed and released. Although, assessing and quantifying the quality of the completed software systems will result in the most defined measurements. However, this framework was developed for offline service recommendation other than software product design. Although the approach and CSTrust framework evaluate the trustworthiness of cloud service, both of them don’t discuss the composite service clearly. Mohammed Elhag and Mohamad, et al.[36] propose a set of metrics to measure the quality of service-oriented design. However, this work can be extended using ComSDM method to propose new metrics or a quality measurement model for composite services. For instance, to calculate the number of operations, services or specific type of relationship, from service-oriented design, one can use this method to collect this information easily by counting all the shapes for each element in the design. This way of representing the different elements in the system facilitate the measuring the quality of design by labeling the providers and consumers and weighted the system elements according to their importance. As a result, many new relationships are identified and represented by new notations using graph theory in order to facilitate the measurement of service-oriented design later on.

The service-oriented modeling method for service-oriented proposed in Gao, et al.[39]is particularly for e-commerce platform. This method describesthe process of the modeling in three phases that are requirement analysis, service-oriented analysis and service-oriented design. However, the method provides a general view about modeling and it is not considered how to do the modeling.

A step-by-step modeling for service-oriented design is missing from[3, 27, 29, 38, 39]and their focus is on what to do rather than how to do it. Conversely, this work proposes a systematic method for modeling composite service through explaining ‘How’ part and gives a step-by-step guideline to model the design of service-oriented using graph theory. Furthermore, the ComSDM method does not only depend on mathematical representation of the components of service-oriented design, but it considers the graph-theory torepresent the components and relationships among the design artifacts.

Among the methods proposed for service modeling there are two approaches to identify the services: Top-Down approach (analyzing the business requirements) and Bottom-Up (existing information system). Moreover, in almost all the modeling methods for service-orienteddesign the identification of services follows the business process. For example, the service identification in [39] is based on the business process modeling. However, the ComSDM method used previously presented service identification guideline that is based on device-centric and task-centriccriteria to group the related operations in the appropriate services. Furthermore, all of the modeling methods for service-oriented deal with enterprise system development and they do not consider devices. Conversely, ComSDM method deal with enterprise system and DERTS and consider the devices.

## Conclusion

This work proposed a step-by-step method for composite service design modelingwhile keeping the service composition considerations in mind. After services are modeled, it is easy to complete the other activities of service-oriented development, such as quality measurement ofthe design phase. The ComSDM method usedpreviously presented service identification guideline and provides the graph-theory basedrepresentation of the components of composite service in service-oriented design. Furthermore, the ComSDM method provides the mathematical representation of the components of service-oriented design using the graph-based theory. The method wassuccessfully implemented in a smart home case studyto show its applicability and to prove itssuitabilityfor the composite service design of distributed embedded real-time system along with enterprise software development. In addition, the ComSDM method validated through measuring the reusability and complexity of system and compared with FMSOD after successfully applying them to smart home. The ComSDM method increased the reusability of service-oriented design and decreased the complexity. Some of the existing techniques for service design modeling are compared with theComSDM method. After the successful modeling of the composite service design, the next step would be the quality measurement of the composite service design.
